# Ultra-Deep Massive Parallel Sequencing of Plasma Cell-Free DNA Enables Large-Scale Profiling of Driver Mutations in Vietnamese Patients With Advanced Non-Small Cell Lung Cancer

**DOI:** 10.3389/fonc.2020.01351

**Published:** 2020-08-04

**Authors:** Le Son Tran, Quynh-Tho Thi Nguyen, Chu Van Nguyen, Vu-Uyen Tran, Thai-Hoa Thi Nguyen, Ha Thu Le, Mai-Lan Thi Nguyen, Vu Thuong Le, Lam-Son Pham, Binh Thanh Vo, Anh-Thu Huynh Dang, Luan Thanh Nguyen, Thien-Chi Van Nguyen, Hong-Anh Thi Pham, Thanh-Truong Tran, Long Hung Nguyen, Thanh-Thanh Thi Nguyen, Kim-Huong Thi Nguyen, Yen-Vi Vu, Nguyen Huu Nguyen, Vinh-Quang Bui, Hai-Ha Bui, Thanh-Thuy Thi Do, Nien Vinh Lam, Kiet Truong Dinh, Minh-Duy Phan, Hoai-Nghia Nguyen, Hoa Giang

**Affiliations:** ^1^Gene Solutions, Ho Chi Minh City, Vietnam; ^2^Medical Genetics Institute, Ho Chi Minh City, Vietnam; ^3^Vietnam National Cancer Hospital, Hanoi, Vietnam; ^4^Ha Noi Oncology Hospital, Hanoi, Vietnam; ^5^Cho Ray Hospital, Ho Chi Minh City, Vietnam; ^6^University of Medicine and Pharmacy at Ho Chi Minh City, Ho Chi Minh City, Vietnam; ^7^HT Genetics, Ho Chi Minh City, Vietnam

**Keywords:** liquid biopsy, circulating tumor DNA, non-small cell lung cancer, actionable mutations, ultra-deep sequencing, tissue biopsy, targeted therapy

## Abstract

Population-specific profiling of mutations in cancer genes is of critical importance for the understanding of cancer biology in general as well as the establishment of optimal diagnostics and treatment guidelines for that particular population. Although genetic analysis of tumor tissue is often used to detect mutations in cancer genes, the invasiveness and limited accessibility hinders its application in large-scale population studies. Here, we used ultra-deep massive parallel sequencing of plasma cell free DNA (cfDNA) to identify the mutation profiles of 265 Vietnamese patients with advanced non-small cell lung cancer (NSCLC). Compared to a cohort of advanced NSCLC patients characterized by sequencing of tissue samples, cfDNA genomic testing, despite lower mutation detection rates, was able to detect major mutations in tested driver genes that reflected similar mutation composition and distribution pattern, as well as major associations between mutation prevalence and clinical features. In conclusion, ultra-deep sequencing of plasma cfDNA represents an alternative approach for population-wide genetic profiling of cancer genes where recruitment of patients is limited to the accessibility of tumor tissue site.

## Introduction

Comprehensive profiling of actionable mutations in non-small cell lung cancer (NSCLC) is vital to precision therapy (1, 2). Therefore, mutation profiling of major driver genes such as *EGFR, KRAS, BRAF, NRAS, ALK*, and *ROS1* has been recommended by the American Society of Clinical Oncology (ASCO) and National Comprehensive Cancer Network (NCCN) for patients with advanced NSCLC (3, 4). Currently, clinical practice guidelines on NSCLC are largely based on results from studies of Caucasian cohorts (4). However, there is a growing body of clinical evidence that NSCLC patients present heterogeneous genetic constitution across different populations, suggesting that large-scale population-specific mutation profiles are of utmost importance to the development of clinical practice guidelines for a particular population (5–7).

Although genetic testing of tumor tissue is the standard method for mutation profiling of NSCLC, the tumor biopsy is invasive and limited by accessibility. In deed, the feasibility of performing tissue biopsy is low in advanced NSCLC patients who are in metastatic stages (8). Consequently, databases built from tissue based testing may under-represent the mutation profiles of advanced NSCLC patients (9). The analysis of cell free DNA (cfDNA) in plasma, known as liquid biopsy, has recently emerged as an alternative and noninvasive approach for exploring tumor genetic landscape (10). This approach involves detecting genetic alterations in circulating tumor DNA (ctDNA), which are 160–200 bp DNA fragments released into the blood circulation by tumor cells undergoing cell death and comprise a fraction of cfDNA (10, 11). The cfDNA based approach allows sample collection for patients at all stages and high-throughput sample processing (12). It is therefore more suitable for population-scale study involving a large number of samples. Additionally, it has been reported that ultra-deep sequencing of plasma cfDNA of NSCLC patients achieved high concordance rates of driver mutation detection compared to sequencing of matched tumor tissues (13–16).

We have previously conducted a study comparing the performance of liquid biopsy and tissue biopsy using matched tissues and plasma samples obtained from the same cohort of patients (15). We observed a high concordance rate of 85% between actionable mutation profiles detected from paired plasma and tissue samples in a cohort of 40 NSCLC patients, consistent with other similar studies (15, 17, 18). However, the major limitation of such studies is that the conclusion was drawn from a relatively small sample size due to the difficulties in obtaining tissue biopsies from NSCLC patients with metastatic cancer. As such, the clinical validation of whether cfDNA sequencing could serve as a non-invasive, alternative approach to tissue biopsy in a larger cohort of patients is needed. However, it has been reported that 20–50% of patients with advanced metastatic NSCLC are not fit for tumor biopsy testing (8, 19), thus making a large-scale comparison between matched plasma and tissue samples a challenge, both logistically and ethically. In this study, we performed liquid biopsy on a large cohort of Vietnamese NSCLC patients to examine whether it can accurately represent the mutation profiles previously obtained by direct analysis of tumor derived DNA (7).

## Results

### Clinical Features of cfDNA and ttDNA Cohorts

This study compared two cohorts with matched clinical features including tumor stages, treatment status, male to female ratio and age. The first cohort, hereafter called cfDNA cohort, included plasma samples collected from a total of 265 treatment-naïve patients with advanced stage NSCLC (stage III-IV) from four different hospitals in Vietnam. The second cohort, hereafter called ttDNA cohort, comprised of 285 tissue samples from Vietnamese NSCLC selected to match the tumor stages and treatment status of the cfDNA cohort. The mutation profiles of this cohort were retrieved from our previously published study (7). Descriptive statistics of these two cohorts showed that, in addition to tumor stages and treatment status, they were also comparable with respect to male-to-female ratio and median age (Table S1). Furthermore, adenocarcinoma (AC) was the dominant histological subtypes (over 90%) among patients with known histology information in both cohorts (Table S1).

### Comparing Mutation Profiles of cfDNA Cohort With Those of ttDNA Cohort

**Figure 1:**
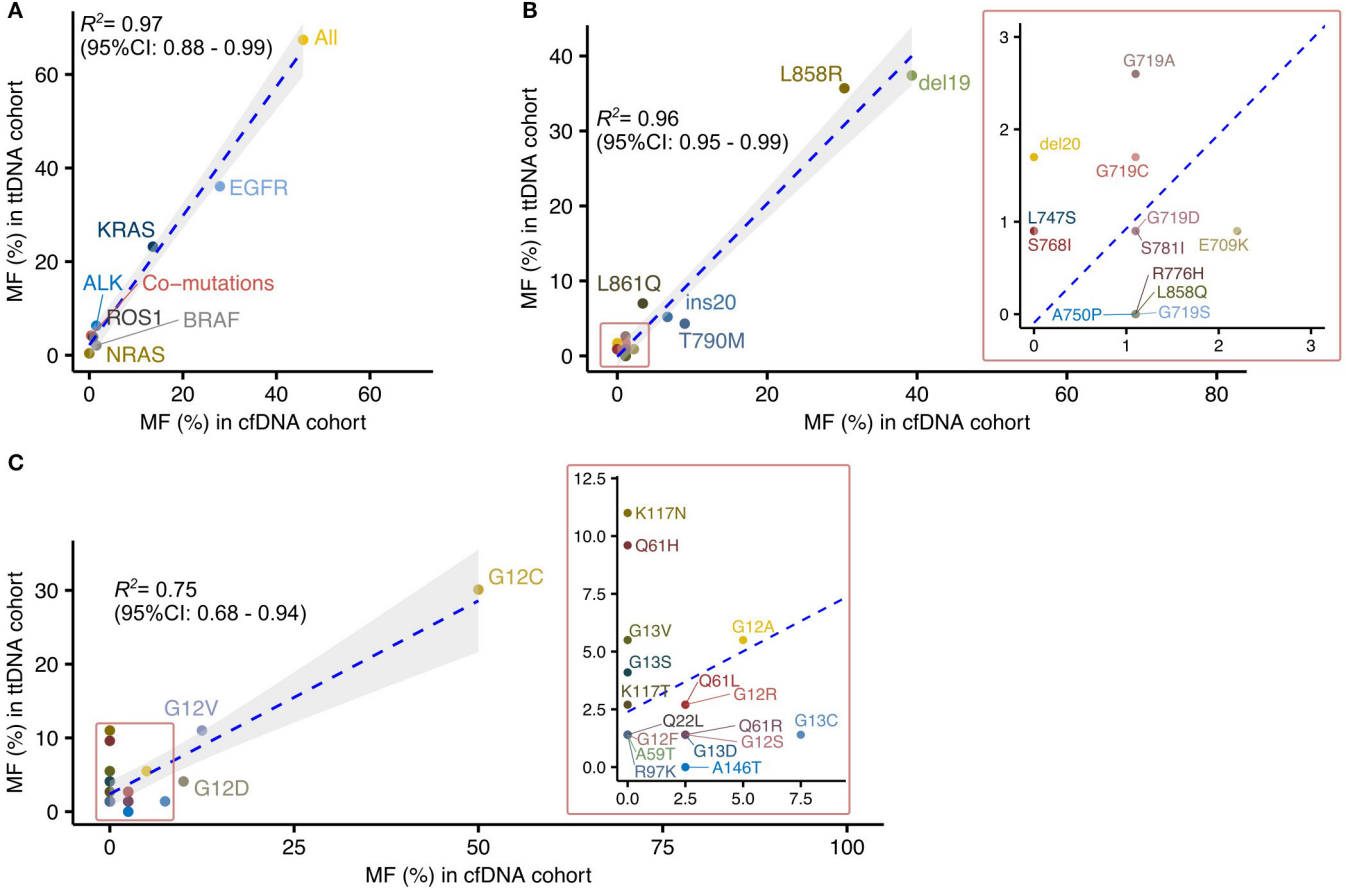
Large-scale sequencing of plasma cfDNA provides mutational profiles highly correlated to those defined by sequencing tumor tissued derived DNA in Vietnamese patients with non-small cell lung cancer. **(A)** Correlation analysis of driver mutation frequencies between cfDNA and ttDNA testing. **(B,C)** Correlated distribution of *EGFR* **(B)** and *KRAS* **(C)** mutation subtypes between cfDNA and ttDNA cohorts.

Among 265 patients in the cfDNA cohort, 121 cases (45.7%) had at least one clinically relevant genetic alteration (according to ClinVar) in the six tested genes (Table S2). This detection rate was significantly lower than the detection rate of 67.4% in ttDNA cohort (Figure 1A, *p* < 0.00001). Likewise, the frequency of cases carrying mutations in two or more tested genes in cfDNA was significantly lower than that of ttDNA (0.4 vs. 4.2%, *p* < 0.05). Mutation frequencies (MF) in the tested genes were all significantly lower in cfDNA cohort compared to ttDNA cohort. There was no mutations detected in *NRAS* in cfDNA cohort compared to a low rate of 0.4% in ttDNA cohort.

There was a significant linear correlation of mutation frequency in each tested gene between the two cohorts (*R*^2^ = 0.97, 95% CI: 0.88–0.99) (Figure 1A). Among the tested genes, *EGFR* and *KRAS* were identified as the two most frequently mutated driver genes in both cohorts, with more than 50% of cases positive for mutations in either genes (Figure 1A). *BRAF* mutations, fusion mutations in *ALK* and *ROS1* were detected in both cohorts at lower frequencies than *EGFR* and *KRAS* mutations (Figure 1B). These data suggested that mutation analysis using cfDNA, despite its lower detection rate, was able to revealed the relative composition of driver mutations within a cohort as comparable to ttDNA analysis.

We next focused our comparison on the mutation profiles of the two most frequently mutated genes, *EGFR* and *KRAS*, between the two cohorts. The frequencies of mutation subtypes in *EGFR* showed high correlation between the two cohorts (*R*^2^ = 0.96, 95% CI 0.95–0.99; Figure 1B). The majority of *EGFR* mutation subtypes (10/13, 76.9%) detected in ttDNA cohorts were also found in cfDNA cohort (Figure 1B). Of those, Del19 and L858R were identified as the dominant subtypes, accounting for more than 70% of all *EGFR* mutations in both cohorts (Figure 1B). Three rare *EGFR* mutation subtypes including S781I, L747S, and del20 with frequencies of 0.9, 0.9, 1.7%, respectively, in ttDNA cohort were not detected in cfDNA cohort. In contrast, certain subtypes including L858Q, R776H, A750P, and G719S were detected in cfDNA cohort but not present in ttDNA cohort (Figure 1B). For *KRAS* mutation subtypes, their frequencies showed lower correlation between the two cohorts than *EGFR* mutations (*R*^2^ = 0.75, 95% CI 0.68–0.94). Our cfDNA analysis detected 10 out of the 19 subtypes (52.3%) present in ttDNA cohort. Of those subtypes, mutations in exon 2 (G12), accounted for the dominant mutation subtypes in both cohorts (Figure 1C). Among the 9 discordant subtypes, four (Q22L, A59T, R97K, K117T) had frequencies of below 2%, while five subtypes (Q61H, K117N, K117T, G13V, and G13S) were present at greater frequencies than this threshold in ttDNA cohort. There was only 1 subtype A146T detected in cfDNA at 2.5% but not present in ttDNA cohort.

Overall, our data suggested that, despite lower detection rate, cfDNA analysis could estimate the composition and distribution patterns of major mutation subtypes compared to those previously defined by massively parallel sequencing of tumor tissues.

### Associations Between Mutation Prevalence and Clinical Features

Previous mutation analysis of tumor tissue samples reported significant associations between certain clinical features among Vietnamese NSCLC patients and the prevalence of *EGFR* and *KRAS* mutations (7). Here, we sought to examine whether cfDNA testing is able to detect such features. We consistently found in both cohorts that *KRAS* mutations were more commonly detected in male patients (*p* < 0.0001, Table 1) while *EGFR* mutations was more frequently found in female (*p* < 0.00001, Table 1). Furthermore, the association between smoking status and mutation frequencies of *KRAS* and *EGFR* showed similar patterns between cfDNA and ttDNA cohort. Specifically, non-smokers showed significantly higher frequency of *EGFR* mutation (*p* < 0.00001, Table 1) but lower frequency of *KRAS* mutation (*p* < 0.00001, Table 1) than smokers. Additionally, it was noted that young patients (≤ 62 years) in cfDNA cohort have significant higher frequency of *EGFR* mutation than older patients (>62 years) (35.9 vs. 21.2%, *p* < 0.05) and such association was not detected in ttDNA cohort. Taken together cfDNA analysis was able to detect unique associations between mutation prevalence and clinical features.

**Table 1:** Association between clinical factors and mutation frequencies of NSCLC driver genes in cfDNA and ttDNA cohorts.

**Cohort**	**Clinical characteristic**		**Total**	***EGFR***				***KRAS***			
				**WT**	**Mutation**	**%**	***p***	**WT**	**Mutation**	**%**	***P***
cfDNA	Sex	Female	100	55	45	**45.0**	<0.00001	97	3	**3.0**	<0.0001
		Male	158	131	27	**17.1**		125	33	**20.9**	
	Age	≤ Median	128	82	46	**35.9**	<0.05	107	21	16.4	>0.05
		>Median	137	108	29	**21.2**		121	16	11.7	
	Smoking status	Yes	144	119	25	**17.4**	<0.00001	113	31	**21.5**	<0.00001
		No	115	66	49	**42.6**		113	2	**1.7**	
ttDNA	Sex	Female	110	54	56	**50.9**	<0.0001	101	9	**8.2**	<0.00001
		Male	174	127	47	**27.0**		117	57	**32.8**	
	Age	≤ Median	145	93	52	35.9	>0.05	113	32	22.1	>0.05
		>Median	139	88	51	36.7		105	34	24.5	
	Smoking status	Yes	43	29	14	**32.6**	<0.05	32	11	**25.6**	<0.05
		No	111	52	59	**53.2**		103	8	**7.2**	

*%: percentage of particular cases in total number cases*.

*Chi squared (χ^2^) test (sample size > 5) was performed to estimate p-value*.

*Bold values are statistically significant (p < 0.05)*.

Since gender, age and smoking status significantly correlated with *EGFR* and *KRAS* mutation frequency in univariate analysis, we selected 252 patients with complete clinical information from the cfDNA cohort for subsequent multivariate analysis to identify significant predictors of mutation frequency. Multivariate logistic regression analysis confirmed gender (*p* = 0.026, odd ratio: 2.86, 95% CI: 1.09–7.51) and age (*p* = 0.043, odd ratio: 0.56, 95% CI: 0.31–1.01) but not smoking status (*p* = 0.47, odd ratio: 0.68, 95% CI: 0.25–1.80) as independent predictors of *EGFR* mutations, whereas smoking status was the only independent predictor of *KRAS* mutations (*p* = 0.025, odd ratio: 7.71, 95% CI: 1.28–46.1) (Table 2). In fact, we did not observed the association between smoking and *EGFR* mutation status when results were stratified by either gender or age, whereas smoking status was consistently associated with *KRAS* status regardless of gender or age (Figure S1). Taken together, our data showed that smoking status is the key predictor for *KRAS* mutations while gender and age together are significant predictors for *EGFR* mutations.

**Table 2:** Multivariate logistic regression analysis for *EGFR* and *KRAS* mutation status in cfDNA cohort.

**Variables**	**Contrast**	***EGFR***				***KRAS***			
		**Regression coefficient**	**SE**	**Odd ratios (95% CI)**	***p*-value**	**Regression coefficient**	**SE**	**Odd ratios (95% CI)**	***p*-value**
Gender	Female vs. Male	0.65	0.29	2.86 (1.09–7.51)	0.026	−0.47	0.92	0.62 (0.10–3.82)	0.612
Age	≤ 62 vs. >62 years	−0.36	0.18	0.56 (0.31–1.01)	0.043	−0.30	0.39	0.74 (0.34–1.58)	0.442
Smoking status	Nonsmokers vs. smokers	−0.21	0.29	0.68 (0.25–1.80)	0.471	2.04	0.91	7.71 (1.28–46.1)	0.025

*A total of 252 patients with complete clinical information in cfDNA cohort were included in the regression analysis. CI, confidence interval; SE, standard error*.

## Discussion

Population-specific profiling of mutations in oncogenes is of critical importance for the understanding of cancer biology in general as well as the establishment of optimal diagnostics and treatment guidelines for that particular population (4). It has been reported that in patients with advanced NSCLC, particularly those with metastatic tumor, the feasibility of performing tissue biopsy is low due to its invasiveness and limited access to tissue sites (8, 20). As such, mutation profiles identified by tumor tissue genetic testing might not precisely represent the heterogeneity of the mutation landscape in a cohort of patients with advanced cancer stages (9). Recently, liquid biopsy, genetic testing of plasma cfDNA shed by tumor cells into the blood stream, has been suggested as a promising approach not only to offer noninvasive testing to advanced stage patients but also to provide more comprehensive picture of mutation profiles due to its unbiased sampling of ctDNA in the blood stream (10, 13). In this study, we evaluated the application of liquid biopsy in a large-scale profiling of population-specific mutation profile by comparing the mutation profiles of six driver genes of 265 Vietnamese patients with advance stage NSCLC defined by sequencing of cfDNA to the existing tissue based sequencing data generated from an independent cohort consisting of 285 patients with similar race, histology and treatment status (7).

We found that the overall mutation frequencies in six tested genes were significant lower in cfDNA cohort compared to cfDNA cohort. There were at least two possible explanations for the lower detection rate by cfDNA testing compared to that of tumor tissue testing. Indeed, previous cross-platform comparison between cfDNA testing and digital PCR reported a sensitivity of cfDNA sequencing of 79% for mutation detection in plasma, suggesting that mutations in cfDNA could not be detected in 20% of the cases (15). This was consistent with findings from previous studies that cfDNA based sequencing exhibited lower sensitivity in mutation detection compared to matched tissue sequencing (14, 15, 20–22). Indeed, cfDNA contains DNA from both normal and cancer cells, with the latter being found at lower abundance and in much more degraded fragments, leading to lower sensitivity in detecting tumor specific mutations (23, 24). Thus, the evidence puts more weight toward the lower detection rate of cfDNA sequencing. Additionally, the lower detection rate could be due to the fact that certain tumor clones might not shed DNA into the circulation.

Despite the lower detection rates across tested genes, the mutation profile established by cfDNA analysis showed similar composition and distribution patterns of mutation subtypes compared to the profile previously generated by tumor tissue sequencing of patients in an independent cohort. Specifically, *EGFR* and *KRAS* were consistently identified as the two most frequently mutated genes, followed by *ALK, ROS1*, and *BRAF*.

We and others have reported high concordance rates between liquid and tissue biopsy testing by comparing mutation profiles of matched plasma and paired tissue samples. However, the major limitation of such studies is that the comparison was mostly carried out in a small cohort of <50 patients due to the difficulties in obtaining sufficient number of matched plasma and tissue biopsies (15, 17, 18). Hence, the analysis was mostly limited to the most commonly mutated driver gene (*EGFR* or *KRAS*). In deed, a large number of mutation subtypes in *EGFR, KRAS* as well as other therapeutically targeted driver mutations such as *ALK* and *ROS1* fusion with low frequencies might be missed and not be taken into account in matched-sample studies. As such, it is essential perform validation in a larger cohort to address whether liquid biopsy can accurately capture the complete mutation profiles of tumor tissues and provide novel insight into the heterogeneity of NSCLC tumor. In fact, we showed that both liquid biopsy and tissue biopsy analysis consistently capture multiple rare subtypes of *EGFR* and *KRAS* in addition to the dominant mutation subtypes of *EGFR* (del19 and L858R) and *KRAS* (codon 12 exon 2). Of those, mutations including T790M and ins20, known to confer resistance to currently available targeted drugs, were consistently detected in the Vietnamese cohort by both biopsy and tissue testing (25). Thus, these finding are novel and clinically significant as they further supported the potential use of liquid biopsy to identify low abundance primary resistance mutation profiles in a particular community, which is important for providing optimal treatment and diagnostic guidelines. It was noted that certain *KRAS* subtypes (K117N, Q61H, and G13V) displayed inconsistent results between two analysis approaches. For example, substitution mutation at codon 117 (K117N) was present in ttDNA of eight cases (11% among all *KRAS* subtypes) but not detected in any case of cfDNA cohort. The most likely explanation for this discrepancy is that tumor cells carrying this type of mutation tend to shed less DNA into the bloodstream, making it below the limit of detection of the current sequencing platform (26, 27). Alternatively, certain tumors were shown to not shed DNA into the circulation (26). On the other hand, there were mutation subtypes of *EGFR* (A750P, L858Q, R776H, and G719S) and *KRAS* (A146T) detected by cfDNA but not present in ttDNA cohort. It is possible that those rare subtypes present at low frequencies in cfDNA cohort might be missed by the insufficient of number of tested cases in ttDNA cohort. Alternatively, they may be derived from somatic mutations arising from the clonal hematopoiesis (28). Thus, future studies are required to examine the origin of these genomic alterations.

We also demonstrated that mutation profiling by cfDNA analysis consistently captured major associations between mutation prevalence and clinical features in Vietnamese cohorts. Thus, these findings suggest that cfDNA based sequencing was capable of identifying and charactering major mutations in driver genes of a NSLCL population. Consistent to previous studies (29, 30), in the present study we identified smoking status as being the key predictor for *KRAS* mutations while gender and age together are significant predictors for *EGFR* mutations in Vietnamese NSCLC patients.

One major limitation of this study is the lack of complete clinical information. For half of patients in ttDNA cohort, smoking status was not recorded. Additionally, half of the patients in cfDNA cohort lacked histology information. In fact, the lack of histological information of patients in the cfDNA cohort truly reflected the clinical context of our study in that tissue biopsy samples were not obtainable in a large proportion of patients with advanced metastatic cancer due to high risk of complication. Previous studies have reported a range of 20–50% of advanced NSCLC patients without tissue biopsy (8, 19). In addition, previous clinical studies consistently reported that stage IIIB or IV adenocarcinoma is the dominant histology (>80%) among Vietnamese NSCLC patients (30, 31). Thus, despite of the missing histology information, we believed that the patients in both cohorts had comparable histology features with majority had advanced adenocarcinoma, minimizing the confounding effect of histology information to our comparison. In deed, our analysis focused on patients diagnosed with advanced stages (III-IV) and naïve to treatments and may not reflect the mutation profiles of NSCLC patients who are in early cancer stages.

In conclusion, we showed that cfDNA genomic testing was able to detect major mutations in tested driver genes that reflected similar mutation composition and distribution pattern, as well as major associations between mutation prevalence and clinical features when compared to those obtained by the tumor tissue based approach. However, the lower detection rate remains a disadvantage of this method that warrants further development. Given the trade-off between sensitivity and non-invasiveness, cfDNA genomic testing is still a suitable tool for large-scale population study where recruitment of patients is not limited to the accessibility of tumor tissue site.

## Materials and Methods

### Patient Recruitments

A total of 265 patients diagnosed with advanced NSCLC (stage IIIB and IV) from Vietnam National Cancer hospital and Ha Noi Oncology hospital, were recruited to this study. Plasma samples were collected from all patients prior to receiving any treatment (cfDNA cohort). Comprehensive patients' clinical information were summarized (Table S1). This study was approved by the Ethic Committee of University of Medicine and Pharmacy at Ho Chi Minh City, Vietnam (Ethic number: 164/HDDD). All patients whose samples were analyzed in this study have provided written consent.

The ttDNA cohort comprised of tissue samples from 285 Vietnamese NSCLC patients selected from our previous study (7). All patients in ttDNA cohort are naïve to treatment and diagnosed with advanced NSCLC at the time of hospital admission.

### Plasma Cell Free DNA Isolation

Ten-milliliter of peripheral blood was drawn in Streck tubes (Cell-free DNA BCT, Streck) and undergone 2 rounds of centrifugation (2,000 × g for 10 min then 16,000 × g for 10 min) to separate plasma from blood cells. The plasma fractions (4–6 mL) were then collected, aliquoted (2 mL per aliquot) and stored at −80°C until cell free DNA extraction.

Cell free DNA was extracted from an aliquot of 2 ml of plasma using the MagMAX Cell-Free DNA Isolation kit (Thermo Fisher, USA) following the manufacturer's instructions.

### Ultra-Deep Massively Parallel Sequencing (MPS) With Unique Molecular Identifier Tagging

Cell free DNA was prepared and sequenced as previously described. Briefly, library with unique molecular identifier tagging was prepared from 2 ng of cfDNA using the Accel-NGS 2S Plus DNA library kit (Swift Biosciences, USA) following the manufacturer's instructions. Library concentrations were quantified using QuantiFluor dsDNA system (Promega, USA). Equal amounts of libraries (150 ng per sample) were pooled together and hybridized with xGen Lockdown probes for six targeted genes *EGFR, KRAS, NRAS, BRAF, ALK*, and *ROS1* (IDT DNA, USA). For *ALK*, and *ROS1*, customized probes for intron regions were designed and mixed with probes for exon regions at equal concentration. Sequencing was run using NextSeq 500/550 High output kits v2 (150 cycles) on Illumina NextSeq 550 system (Illumina, USA) with minimum target coverage of 10,000 X. A total on-target fraction of 20–40% was detected.

### Variant Calling Using Mutect2

Each sample was barcoded with a single 8-bp index in the P7 primer and each DNA fragment were tagged with a unique identifier consisting of a random 9-bp sequence within the P5 primer. Pair-end (PE) reads and the correspondent unique identifier sequences were generated using bcl2fastq package (Illumina). The reads were aligned to human genome (hg38) using BWA package and then grouped by the unique identifier in order to determine a consensus sequence for each fragment, eliminating sequencing and PCR errors that account for <50% of reads per fragment. The consensus reads were used for final variant calling using Mutect2 (32). A custom pipeline with call to BWA 0.7.1 (33), Picard 2.18.23 (Broad Institute, GitHub Repository), Samtools 1.9 (33) and Fulcrum genomic analysis packages (34) were built to perform the above-mentioned analysis steps. Unique molecular identifier (UMI) grouping was performed by using fgbio 0.8.0 package (Fulcrum Genomics). For detection of ALK and ROS1 rearrangement, fusion variant calling was analyzed using Factera v1.4.4 with default parameters (35).

### Statistical Analysis

Differences in demographic features between cfDNA and ttDNA cohort was compared by Kruskal-Wallis and Dunn's multiple comparison test for age and Fisher's exact test for other variables (Table 1). Pearson's chi-squared (χ^2^) test (sample size > 5) or Fisher's exact test (sample size ≤ 5) was performed on the web page “Social Science Statistics” (http://www.socscistatistics.com) to assess the association between two categorical variables (Table 1). Bonferroni correction was applied when multiple comparisons were performed. Multivariate logistic regression analysis was performed using XLSTAT tool (2020.2.3) was performed to assess the correlation of *EGFR* and *KRAS* mutation with age, gender and smoking status.

## Data Availability Statement

The datasets in this study have been upload to the NCBI SRA repository (https://www.ncbi.nlm.nih.gov/bioproject). The accession number is PRJNA642002.

## Ethics Statement

This study was approved by the Ethic Committee of University of Medicine and Pharmacy at Ho Chi Minh City, Vietnam (Ethic number: 164/HDDD). The patients/participants provided their written informed consent to participate in this study.

## Author Contributions

V-UT, BV, A-TD, LTN, T-CN, H-AP, T-TT, and LHN performed experiments. CN, T-HN, HL, M-LN, VL, and V-QB recruited patients and performed pathological analysis. T-TN, K-HN, Y-VV, NN, H-HB, and L-SP collected and analyzed clinical data. Q-TN, T-TD, NL, and KT designed experiments and analyzed data. M-DP revised the manuscript. H-NN supervised the project, analyzed the data, and wrote the manuscript. HG designed experiments and analyzed sequencing data. All authors contributed to the article and approved the submitted version.

## Conflict of Interest

LT, V-UT, BV, LTN, T-CN, H-AP, T-TT, LHN, T-TN, K-HN, Y-VV, NN, M-DP, and HG were employed by Gene Solutions. The remaining authors declare that the research was conducted in the absence of any commercial or financial relationships that could be construed as a potential conflict of interest.
