# Supplementary figures and images for: Ultra-Deep Massive Parallel Sequencing of Plasma Cell-Free DNA Enables Large-Scale Profiling of Driver Mutations in Vietnamese Patients With Advanced Non-Small Cell Lung Cancer
Source: Front Oncol. 2020 Aug 4;10:1351. doi: 10.3389/fonc.2020.01351 (PMC7418519; doi:10.3389/fonc.2020.01351)

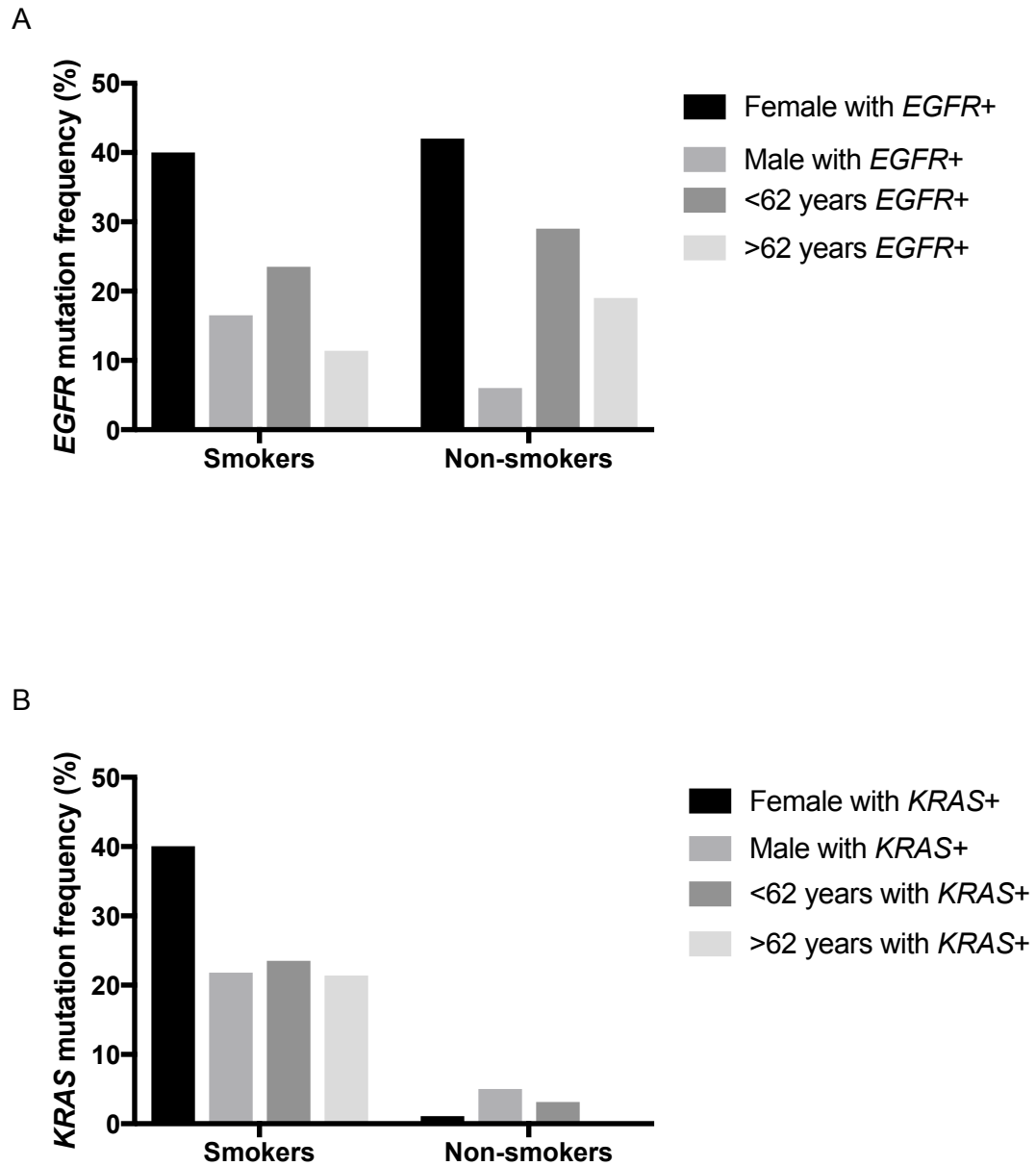

**Figure S1** The effect of smoking status on *EGFR* (A) or *KRAS* (B) mutation frequencies.

Supplement: Supplementary file 3 [file Image_1.pdf]
